# Reply: “Entering a new GALAXI: The importance of long-term data in Crohn’s disease clinical trials”

**DOI:** 10.1093/ibd/izag082

**Published:** 2026-05-15

**Authors:** Anita Afzali, Rian Van Rampelbergh

**Affiliations:** Division of Gastroenterology and Hepatology, University of Cincinnati College of Medicine, Cincinnati, OH, United States; Johnson & Johnson, Antwerp, Belgium

**To the Editors**:

We read with interest the editorial by Sleiman et al on the importance of long-term data in Crohn’s disease clinical trials. We thank the authors for highlighting our recent report of 5-year efficacy and safety data for guselkumab in patients with moderately to severely active Crohn’s disease from the phase 2 GALAXI 1 study.^1^

The long-term extension (LTE) of the GALAXI 1 study provides the longest reported follow-up to date for guselkumab in Crohn’s disease. While limited by the small sample size inherent to a phase 2 study, these data provide important context while awaiting LTE results from the ongoing, phase 3 GALAXI 2 and 3 studies.^2^ These findings should be interpreted within the constraints of the study design, including sample size and the open-label nature of the LTE, and are intended to provide supportive longitudinal insights rather than definitive comparative conclusions.

As highlighted by Sleiman et al, we included both nonresponder imputation and as-observed analyses of the long-term efficacy endpoints to address known limitations of long-term data interpretation, including patient attrition. These complementary approaches address distinct and clinically relevant questions. Nonresponder imputation analysis provides a conservative estimate of treatment effectiveness from initiation of guselkumab, whereas as-observed analyses reflect outcomes among patients remaining on therapy over time. Together, these approaches provide a balanced and methodologically rigorous assessment of long-term treatment performance. Importantly, these complementary analytic approaches provide context for interpreting long-term outcomes in the presence of attrition and help mitigate bias inherent to LTE study designs. Findings should be interpreted within LTE design constraints but may offer relevant insights to inform clinical practice.

Disease-modifying outcomes (ie, abdominal surgeries and hospitalizations) remain a key area of interest, and data on medical resource utilization were collected during long-term follow-up. Hospitalization and emergency care utilization occurred in 29% (n = 53 of 185) of guselkumab-treated participants and 24% (n = 15 of 63) of ustekinumab-randomized participants from week 0 through week 240. Event rates for surgery were low (n = 6 and n = 1, respectively), limiting interpretability for this endpoint. Ongoing phase 3 GALAXI 2 and 3 LTE studies will provide greater clarity on the impact of guselkumab on disease-modifying outcomes in larger populations.

Long-term extensions of clinical trials, including phase 2 studies, are essential to characterize durability of efficacy and safety of Crohn’s disease therapies. The 5-year follow-up data from the GALAXI 1 study provide important context for the primary study findings.^3^^,^^4^ Data from ongoing phase 3 LTE studies will further define the long-term clinical profile of guselkumab.
